# Refractory Cushing’s Disease Complicated by Severe Thrombocytopenia and Pituitary Apoplexy Culminating in a Fatal Outcome: A Case Report

**DOI:** 10.7759/cureus.118034

**Published:** 2026-10-16

**Authors:** Maria Inês Lopes, Gonçalo Magalhães, André Rosa Alexandre, Maria Carolina Paulino, Elisa Vedes, Isabel Esperança, José P Carda, Francisco S Rosario, António Messias

**Affiliations:** 1 Intensive Care Unit, Hospital da Luz Lisboa, Lisbon, PRT; 2 Department of Hematology, Hospital da Luz Lisboa, Lisbon, PRT; 3 Department of Endocrinology, Hospital da Luz Lisboa, Lisbon, PRT

**Keywords:** acth-dependent hypercortisolism, adrenalectomy, cushing's disease, pituitary apoplexy, thrombocytopenia

## Abstract

Hypercortisolemia associated with Cushing’s disease is classically recognized as a hypercoagulable state. Conversely, severe thrombocytopenia and hemorrhagic complications are exceptionally rare presentations.

We report a case of a 59-year-old woman who presented with headache, proximal weakness, and Cushingoid features. Examination revealed severe Cushingoid features. Laboratory findings showed metabolic alkalosis, severe hypokalemia, and mild thrombocytopenia. Biochemical evaluation confirmed adrenocorticotropic hormone (ACTH)-dependent hypercortisolism (cortisol 157 µg/dL, ACTH 212 pg/mL), and a magnetic resonance imaging (MRI) scan revealed a 28-mm invasive pituitary macroadenoma extending into the sphenoid sinus and left cavernous sinus.

The patient was admitted to the intensive care unit for electrolyte replacement and blood pressure control. Thrombocytopenia worsened progressively alongside mucocutaneous bleeding. Diagnostic workup showed preserved megakaryopoiesis, normal ADAMTS-13 activity, absent schistocytes, and elevated factor VIII and von Willebrand factor levels, pointing toward platelet consumption driven by severe endothelial stress.

Given severe tissue fragility and progressive thrombocytopenia, transsphenoidal surgery was deferred, and medical therapy with cabergoline, mifepristone, and metyrapone was initiated. However, dose escalation was halted by worsening hypokalemia and refractory symptoms. The patient developed central diabetes insipidus (DI), necessitating desmopressin.

On day 29, acute neurological deterioration with a left third cranial nerve palsy occurred; repeat neuroimaging confirmed acute pituitary apoplexy. Emergency transsphenoidal decompression was deferred due to profound thrombocytopenia and carotid artery encasement.

Facing refractory disease, salvage bilateral laparoscopic adrenalectomy was performed on day 31. Postoperatively, the patient developed refractory shock, ischemic hepatitis, and anuric acute kidney failure. Despite multimodal medical therapy, she succumbed to multiorgan failure on day 35.

This case illustrates a rare and life-threatening presentation of Cushing’s disease complicated by suspected secondary consumptive thrombocytopenia and sellar apoplexy, highlighting the critical challenges of managing fulminant hypercortisolemia when emergency neurosurgical intervention carries prohibitive operative risks. This case underscores the locally invasive behavior of some pituitary tumors and the need for multidisciplinary management of severe Cushing's disease complications.

## Introduction

Cushing's disease, caused by adrenocorticotropic hormone (ACTH)-secreting pituitary adenomas, is a rare endocrine disorder with an estimated prevalence of 40-79 cases per million. Despite its rarity, the disease carries substantial morbidity and mortality, with approximately 10-20% of cases presenting with aggressive phenotypes characterized by rapid progression, severe metabolic derangements, and resistance to standard therapeutic modalities [1,2].

Endogenous hypercortisolemia induces extensive systemic and metabolic derangements, including severe hypokalemic alkalosis, marked immunosuppression, and vascular fragility. Concurrently, the hematological manifestations of Cushing's disease are typically dominated by a prothrombotic state resulting from endothelial dysfunction, elevated procoagulant factors (von Willebrand factor and factor VIII), and impaired fibrinolysis [3,4]. In stark contrast to this expected hypercoagulable phenotype, the occurrence of severe, progressive thrombocytopenia and spontaneous bleeding manifestations in active Cushing’s disease is extraordinarily rare and paradoxical. Furthermore, acute hemorrhagic infarction within a pituitary tumor (pituitary apoplexy) can critically intersect with this coagulopathy, either precipitated by severe platelet consumption or contributing to secondary hematological decompensation. Currently, the literature on this clinical intersection is primarily limited to isolated case reports, and the underlying pathophysiological mechanisms driving this interplay remain poorly characterized [5,6].

First-line treatment for Cushing's disease is transsphenoidal surgery, achieving remission in 50-90% of cases depending on tumor characteristics [7]. However, in fulminant presentations with massive ACTH-dependent hypercortisolemia, profound metabolic instability, acute bleeding risk, and a locally invasive sellar mass, standard transsphenoidal decompression may carry prohibitive intraoperative risk.

Medical therapy serves as preoperative optimization or bridge-to-surgery management, while bilateral adrenalectomy represents a definitive option for refractory disease, providing immediate cessation of cortisol production.

We report a fatal case of fulminant Cushing’s disease caused by an invasive, rapidly progressive ACTH-secreting pituitary macroadenoma, in which aggressive tumor growth and intrinsic vascular fragility precipitated pituitary apoplexy alongside severe, paradoxical consumptive thrombocytopenia. This report illustrates the intricate interplay between tumor invasiveness, acute apoplexy, and platelet consumption, highlighting the profound clinical challenges posed by invasive corticotroph tumors.

## Case presentation

A 59-year-old woman presented to the emergency department with an eight-month history of persistent bitemporal headaches (rated 7/10 in intensity), associated with proximal muscle weakness, lower limb edema, and back pain following a slight fall.

Her medical history included hypertension and hypothyroidism. Physical examination revealed hypertension (blood pressure 190/118 mmHg) and a Cushingoid phenotype with buffalo hump, facial plethora, multiple petechiae, purpura, and violaceous striae distributed across the trunk and extremities. Low back pain was present (rated 8/10 in intensity), without radiation, and relieved by rest.

Laboratory analysis revealed metabolic alkalemia (pH 7.52; normal range: 7.35-7.45), severe hypokalemia (K+ 1.7 mmol/L; normal range: 3.5-5.0 mmol/L), hypomagnesemia (Mg2+ 1.2 mg/dL; normal range: 1.7-2.2 mg/dL), and hypophosphatemia (PO43- 2.1 mg/dL; normal range: 2.5-4.5 mg/dL). Hematological assessment showed thrombocytopenia (platelets 144 × 10^9^/L; normal range: 150-400 × 10^9^/L) with normal hemoglobin, coagulation times (PT/aPTT), and fibrinogen levels. Human immunodeficiency virus (HIV-1/2 Ag/Ab) and hepatitis B and C virus markers (HBsAg, anti-HBc, and anti-HCV) were negative.

Hormonal profile confirmed ACTH-dependent hypercortisolism, with morning cortisol (157 µg/dL; normal range: 5-25 µg/dL) and ACTH (212 pg/mL; normal range: 3.6-60.5 pg/mL). A high-dose dexamethasone suppression test (8 mg overnight) showed 58% suppression of serum cortisol (65.89 µg/dL). Secondary anterior pituitary deficiencies were identified, including central hypothyroidism (free T4 0.58 ng/dL (reference: 0.93-1.70 ng/dL); TSH 0.76 µIU/mL), requiring levothyroxine replacement, hypogonadotropic hypogonadism (LH 0.2 mIU/mL, FSH 2.8 mIU/mL), and normal prolactinemia (PRL 8.4 ng/mL). On day 1, antidiuretic hormone (ADH) was normal at 3.2 pg/mL (1-3.6 pg/mL).

Despite severe hypertension and profound hypokalemic alkalosis, plasma aldosterone was low-normal (4.08 ng/dL; reference: 3.0-16.0 ng/dL), with suppressed plasma renin activity, excluding primary hyperaldosteronism and indicating a non-aldosterone-mediated mineralocorticoid excess state.

A contrast-enhanced magnetic resonance imaging (MRI) revealed a 28-mm locally invasive suprasellar lesion with inferior and lateral extension, compatible with an invasive macroadenoma. Notably, the usual posterior pituitary bright spot was absent. No significant signal intensity changes were observed in the cerebral, cerebellar, or brainstem parenchyma (Figure 1). Abdominal MRI revealed adrenal glands with slightly bulbous limbs but no focal nodular lesions. Thoracoabdominopelvic computed tomography revealed no pulmonary, thymic, or gastroenteropancreatic neuroendocrine lesions, with no evidence of an ectopic source. A spinal MRI confirmed an osteoporotic fracture of the thoracic spine.

**Figure 1:**
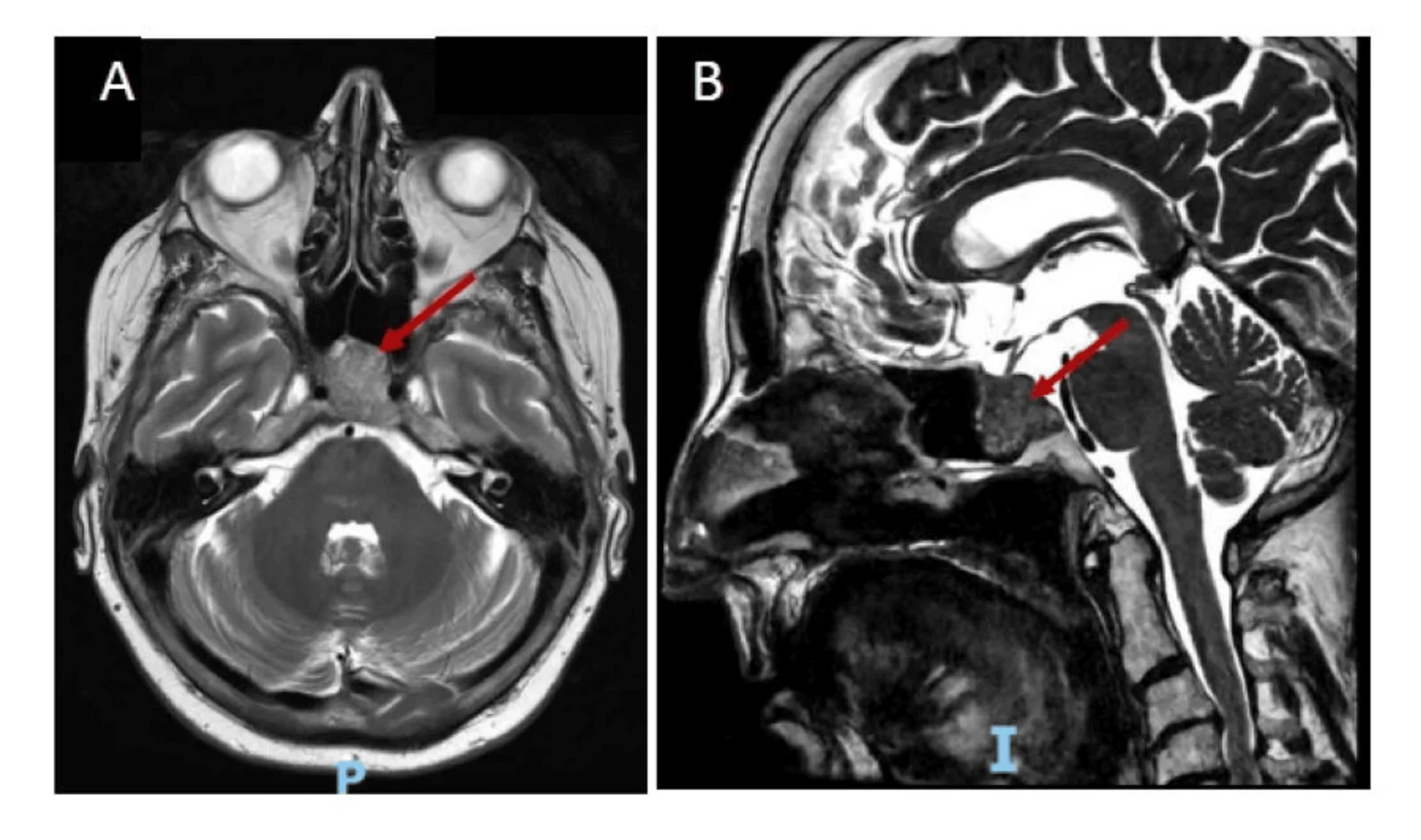
Baseline sellar magnetic resonance imaging (MRI) at presentation. (A) Axial T2-weighted fast spin-echo image at the level of the orbits and pons. It shows a large, heterogeneous sellar mass (28 × 22 × 20 mm) (red arrow). The mass extends anteriorly and inferiorly into the sphenoid sinus and laterally toward the cavernous sinus, predominantly on the left side.(B) Sagittal T2-weighted image. It shows a heterogeneous sellar mass with predominantly inferior extension (red arrow). The mass erodes the sellar floor and dorsum sellae and invades the sphenoid sinus and upper clivus. The suprasellar cistern and optic chiasm are preserved.

The patient was admitted to the intensive care unit for close monitoring. Initial management included continuous potassium infusion and intravenous labetalol for blood pressure control.

At admission, an exuberant mucocutaneous hemorrhagic diathesis was noticed, characterized by spontaneous bleeding at venipuncture and arterial puncture sites, along with progressive petechiae and purpura, despite initially near-normal platelet counts (144 × 10^9^/L). This severe bleeding tendency occurred in the absence of coagulopathy (normal PT, aPTT, and fibrinogen). Over the subsequent days, thrombocytopenia worsened rapidly, dropping from 144 to 105 × 10^9^/L on day 3 and to less than 100 × 10^9^/L on day 8.

Following multidisciplinary evaluation by endocrinology, neurosurgery, and intensive care teams, immediate transsphenoidal surgery was deemed prohibitively high-risk. This clinical decision integrated the severe cortisol-induced vascular and tissue friability, together with rapidly worsening thrombocytopenia. Consequently, initial priority was given to an aggressive, stepped, multimodal medical regimen.

Cabergoline was started at 0.5 mg twice weekly (on day 3) and up-titrated to 7.0 mg weekly (on day 31) as a tumor-directed D2-receptor agonist. Simultaneously, while correcting hypokalemia, off-label use of mifepristone (up to 600 mg daily) was introduced to achieve rapid peripheral glucocorticoid receptor blockade. However, rapid clinical deterioration was observed, reflecting a lack of response to receptor blockade. Consequently, on day 21, the pharmacological strategy was shifted from mifepristone to metyrapone, a potent 11-beta-hydroxylase inhibitor. Metyrapone was titrated to 4,000 mg daily; nonetheless, further dose escalation was impeded by secondary mineralocorticoid accumulation, which exacerbated refractory hypokalemia and severe hypertension.

Metyrapone produced a partial biochemical response, with serum cortisol decreasing from 323 µg/dL on day 27 to 110 µg/dL on day 31, accompanied by a reciprocal rise in ACTH. However, clinical control was not achieved: severe hypokalemic metabolic alkalosis persisted despite continuous high-dose parenteral potassium repletion, hypertension remained uncontrolled, and progressive neuromuscular weakness was followed by acute neurological deterioration. The temporal evolution of serum cortisol (measured by chemiluminescent immunoassay) and ACTH is shown in Figure 2, Panel A. Progressive thrombocytopenia was noticed, worsening to 27 × 10^9^/L, accompanied by hemoglobin 6.6 g/dL (normal range: 12-15.5 g/dL) (Figure 2, Panel B).

**Figure 2:**
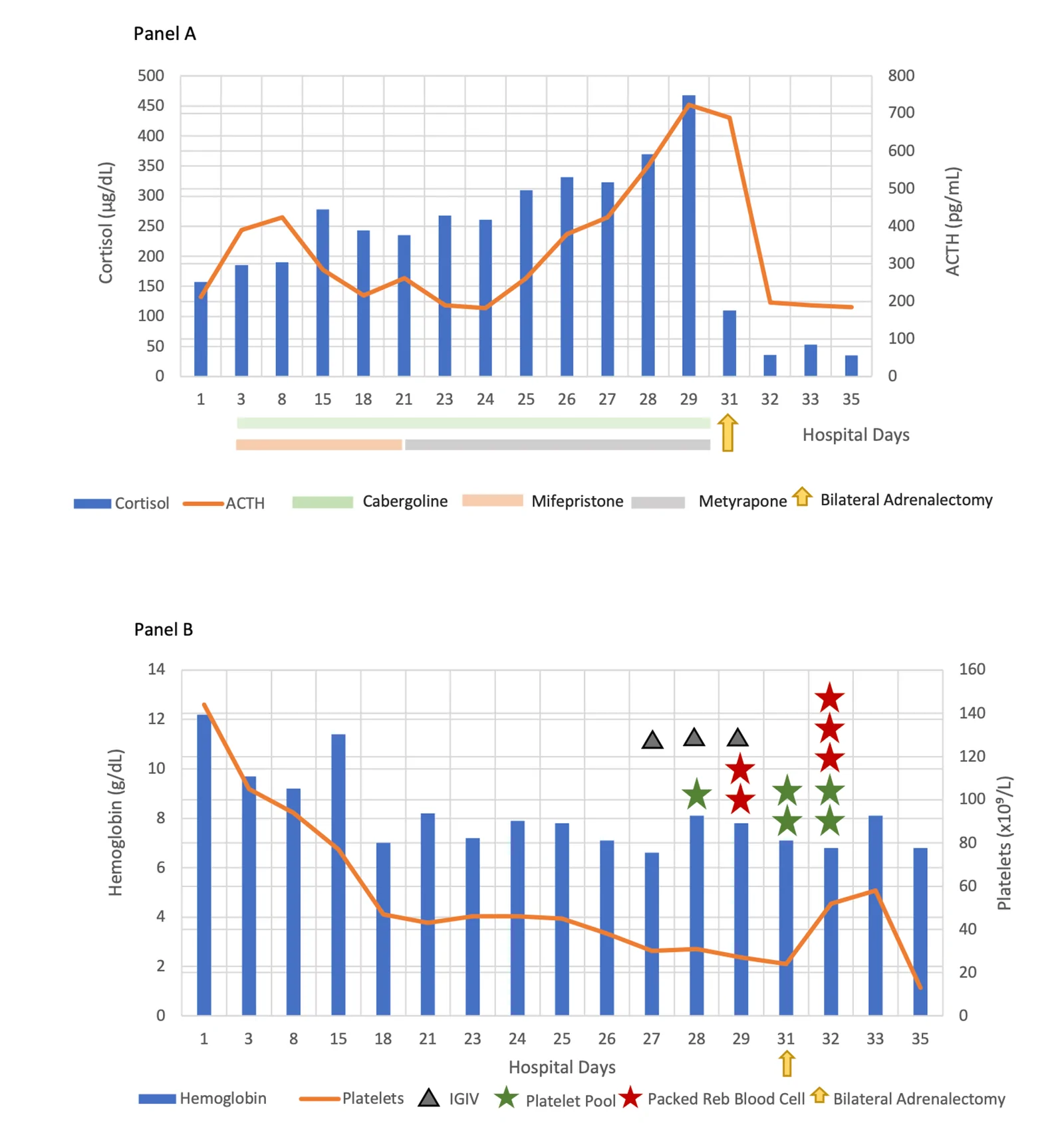
Longitudinal timeline of endocrine, hematological, and therapeutic milestones throughout hospitalization (days 1-35). Panel A (Endocrine trajectory and therapeutic interventions): Temporal profiles of serum adrenocorticotropic hormone (ACTH, pg/mL; orange line) and serum total cortisol (μg/dL; blue column). Horizontal colored bars indicate the duration and timing of medical therapies: cabergoline (days 3-30), mifepristone (days 3-21), and metyrapone (days 21-30). The yellow arrow marks the emergency bilateral adrenalectomy on day 31. Note: Cortisol values during metyrapone therapy may be overestimated because of immunoassay cross-reactivitywith 11-deoxycortisol; post-adrenalectomy values reflect exogenous hydrocortisone. Panel B (Hematological trajectory and transfusion therapy): Temporal profiles of platelet count (×10^9^/L, orange line) and hemoglobin concentration (g/dL, blue column). Key clinical nadirs of platelets are annotated: pituitary apoplexy event (27 × 10^9^/L on day 29), preoperative nadir (24 × 10^9^/L on day 31), and terminal count (13 × 10^9^/L on day 35). Grey triangles indicate Intravenous Immunoglobulin (IVIG); the red star indicates packed red blood cells; the green star indicates platelet pool; and the yellow arrow indicates bilateral adrenalectomy. ACTH: adrenocorticotropic hormone. Y-axis represents concentration; X-axis represents days.

A systematic hematological work-up was performed. Peripheral blood smears demonstrated no red blood cell fragmentation or schistocytes (0%), with a reticulocyte count of 4.7% (normal range: 0.5-1.5%). Elevated lactate dehydrogenase (LDH 438-606 U/L; reference: 135-225 U/L) and reduced haptoglobin level (5 mg/dL; normal range: 30-200 mg/dL) were noted. The direct antiglobulin test (Coombs) was negative. ADAMTS13 activity was 102% (reference: >40.5%).

There was no evidence of overt disseminated intravascular coagulation: fibrinogen was 332-356 mg/dL (reference: 200-400 mg/dL), D-dimer 3.64 mg/L (reference: <0.5 mg/L), with normal PT and aPTT (ISTH DIC score 4). Rotational thromboelastometry (ROTEM®; Werfen, Barcelona, Spain) performed on day 8 (platelet count 94 × 10^9^/L) showed normal clot formation and firmness, with no evidence of hyperfibrinolysis.

Vitamin B12 (365 pg/mL; reference: 211-911 pg/mL) and folate (8.4 ng/mL; reference: >5.4 ng/mL) levels were within normal range, excluding a nutritional cause of bicytopenia. A bone marrow aspirate was performed on day 17 to differentiate central production failure from peripheral destruction (platelet count 50 × 10^9^/L, hemoglobin 7-8 g/dL). Marrow cellularity was normal for age, with normal trilineage hematopoiesis and no dysplasia; megakaryocytes were present in normal numbers with normal morphology. The differential count (200 cells) showed blasts 4.5%, erythroblasts 23%, and a myeloid-to-erythroid ratio of 2.7, with no eosinophils or monocytes. Mature small lymphocytes showed no atypia. These findings excluded a central cause and were consistent with peripheral destruction of platelets and erythrocytes.

Hemophagocytic lymphohistiocytosis was considered unlikely, with ferritin 150 µg/L (reference: 10-291 µg/L), triglycerides 146 mg/dL (reference: <150 mg/dL), and no hemophagocytosis on the bone marrow aspirate (HScore 84 points; <1%).

Additional studies revealed platelet function analysis (PFA-100®; Siemens Healthineers, Erlangen, Germany) performed on day 5 (platelet count 102 × 10^9^/L, hematocrit 31%) showed mildly prolonged closure times with both cartridges: collagen/epinephrine 173 s (reference: 94-165 s); collagen/ADP 129 s (reference: 71-118 s). Serum protein electrophoresis showed no monoclonal component. Heparin-induced thrombocytopenia (HIT) was excluded based on a low pre-test probability (4T score 2) and negative anti-PF4/heparin antibody screening. Antinuclear antibody was negative.

Further investigation revealed a hypercoagulable profile with elevated activity of factor V 237% (normal range: 62-139%), factor VIII 211% (normal range: 70-150%), and von Willebrand factor activity 315% (normal range: 48-173%).

Given progressive thrombocytopenia with active mucocutaneous bleeding, bone marrow findings consistent with peripheral platelet destruction, and exclusion of microangiopathic, drug-induced, and heparin-induced causes, a secondary immune thrombocytopenia was considered. Intravenous immunoglobulin (IVIG) was started on day 27 (platelet count 30 × 10^9^/L) at 1 g/kg/day for three days as a combined therapeutic and diagnostic trial. No sustained platelet response was observed (platelet count after IVIG 27 × 10^9^/L on day 29). In light of this lack of response and of the markedly elevated factor VIII and von Willebrand factor levels, the thrombocytopenia was ultimately interpreted as a suspected secondary consumptive process associated with endothelial activation. Complete laboratory work-up is summarized in Table 1.

**Table 1:** Timeline of hematological, hormonal, and biochemical parameters during hospitalization.

Parameter (reference range)	Day 1	Day 15	Day 21 (metyrapone switch)	Day 27	Day 29 (apoplexy)	Day 31 (surgery)	Day 33	Day 34	Day 35
Cortisol (5.0-25.0 μg/dL)	157	277	235	323	468	110	53	50	34
ACTH (3.6-60.5 pg/mL)	212	284	261	424	722	689	189	184	185
Platelets (150-400 × 10^9^/L)	144	77	43	30	27	24	58	19	13
Hemoglobin (12.0-15.5 g/dL)	12.2	11.4	8.2	6.6	7.8	7.1	8.1	7.6	6.8
Potassium (3.5-5.0 mmol/L)	1.7	2.4	2.8	2.6	2.4	3.1	3.8	3.5	4.2
Sodium (136-145 mEq/L)	140	152 (DI onset)	144	142	141	143	140	138	141
Creatinine (0.5-1.1 mg/dL)	0.6	0.7	0.8	1.1	1.3	1.6	3.8	2.2	2.8
Arterial lactate (<1.3 mmol/L)	0.8	0.6	0.9	1.2	2.1	2.8	10.7	13	15.0
INR (0.8-1.2)	0.88	0.84	0.91	0.92	0.96	0.96	1.66	4.13	6.04
AST (15-37 UI/L)	28	42	72	97	138	243	8,225	10,355	16,194
ALT (14-59 UI/L)	34	65	110	133	158	198	4,316	5,112	6,155
Total bilirubin (<1.0 mg/dL)	0.42	0.48	0.55	0.59	0.63	0.65	2.33	2.71	4.62
Conjugated bilirubin (<0.2 mg/dL)	0.12	0.18	0.22	0.25	0.25	0.26	1.24	1.83	1.73
LDH (135-225 UI/L)	438	503	669	766	870	967	2,419	5,842	20,978

Concurrently, the patient developed transient central diabetes insipidus (DI), manifested by abrupt polyuria and acute hypernatremia, with serum sodium rising from a baseline of 140 mEq/L to a peak of 152 mEq/L. Concomitant urine osmolality was 374 mOsm/kg. Prompt administration of titrated parenteral desmopressin controlled urine output and successfully restored serum sodium concentrations to 138-143 mEq/L.

On day 29, the patient developed acute neurological deterioration, characterized by worsening headaches, diplopia, and left-sided ptosis. Concomitantly, plasma ACTH reached its highest value (722 pg/mL on day 29; Figure 2, Panel A). Repeated brain MRI confirmed a large, heterogeneous sellar mass measuring 30 × 32 × 22 mm, with intrinsic T1 hyperintensity and T2 hypointensity, consistent with hemorrhagic components. A significant mass effect was observed, including obliteration of the suprasellar cistern. The tumor encased the left internal carotid artery (>180°), indicating invasion of the left cavernous sinus (Figure 3).

**Figure 3:**
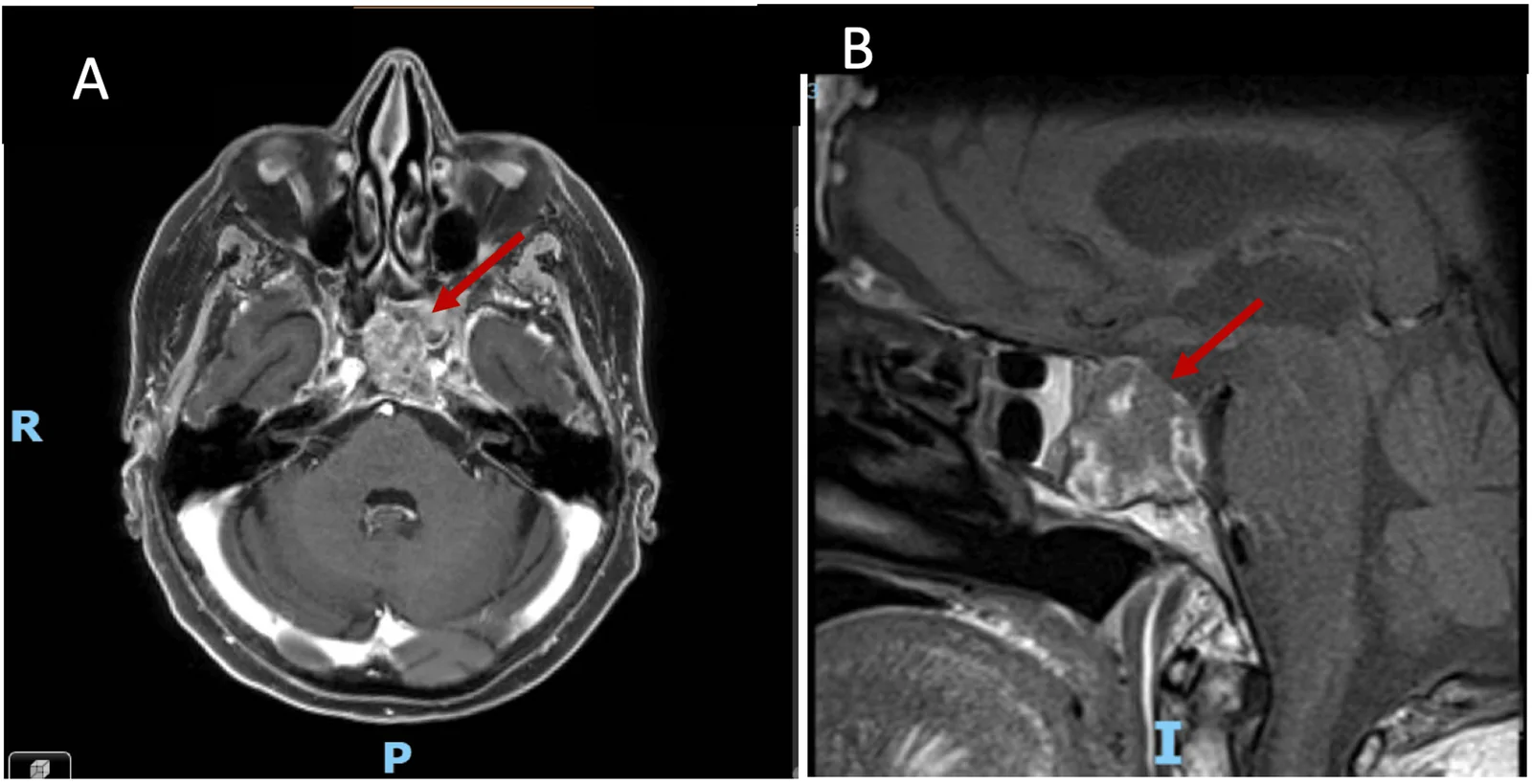
Repeat pituitary MRI on day 29, following acute neurological deterioration. (A) Axial contrast-enhanced, fat-suppressed T1-weighted image. The sellar mass is larger and markedly heterogeneous, with areas of irregular enhancement mixed with non-enhancing foci (red arrow). It extends anteriorly into the sphenoid sinus and laterally toward both cavernous sinuses, predominantly on the left side. (B) Sagittal contrast-enhanced T1-weighted image. It shows a heterogeneous sellar-infrasellar mass with a peripheral enhancing rim and central non-enhancing areas (red arrow). The mass extends into the sphenoid sinus and invades the clivus, reaching the prepontine cistern.

Emergent neurosurgical consultation evaluated the indication for transsphenoidal decompression. However, after multidisciplinary consensus, operative decompression was deemed prohibitively hazardous due to profound consumptive thrombocytopenia (27 × 10^9^/L), posing a catastrophic risk of uncontrollable sellar and intracranial hemorrhage, and extensive invasion of the left cavernous sinus with circumferential encasement of the internal carotid artery, making blind or aggressive debulking lethal in an uncorrectable hemostatic state.

Given the clinical deterioration and the prohibitive intracranial risks, the multidisciplinary team decided to proceed with bilateral laparoscopic adrenalectomy as a definitive treatment. Preoperative optimization included three pooled platelet units and two units of packed red blood cells.

A bilateral laparoscopic adrenalectomy was performed on day 31 without immediate complications. Intraoperatively, noradrenaline was initiated, and two pooled platelet units and three units of packed red blood cells were administered. The patient was readmitted to the ICU sedated and mechanically ventilated.

The postoperative course was marked by refractory shock and multiorgan failure. Hemodynamic monitoring via transpulmonary thermodilution demonstrated severe distributive shock with concomitant cardiac dysfunction. Circulatory support was titrated to a noradrenaline dose of 2 mcg/kg/min, a dobutamine dose of 12.5 mcg/kg/min, and a vasopressin dose of 0.04 U/min. Concurrently, aggressive steroid coverage with stress-dose hydrocortisone (200 mg/day) and fludrocortisone (100 mcg bolus, followed by 50 μg/day) was administered. A thoraco-abdominal CT scan showed no signs of active intra-abdominal hemorrhage or hematoma in the adrenalectomy beds. Histopathological examination of the adrenal glands revealed adrenocortical hyperplasia.

A rapid clinical decline ensued, characterized by refractory multiorgan failure with severe ischemic hepatic necrosis (peak AST 16,194 UI/L, ALT 6,155 UI/L, total bilirubin 4.62 mg/dL, INR 6.04, and arterial lactate 15 mmol/L). Concurrently, stage 3 anuric acute kidney injury developed (serum creatinine 3.8 mg/dL, urea 184 mg/dL) alongside severe, refractory metabolic acidemia (pH < 7.10, HCO₃⁻ < 10 mmol/L), prompting initiation of continuous renal replacement therapy via continuous veno-venous hemodiafiltration (CVVHDF) on postoperative day 1. Circuit anticoagulation was withheld due to severe consumptive thrombocytopenia and fulminant ischemic hepatitis. Thrombocytopenia worsened progressively, reaching a postoperative nadir of 13 × 10^9^/L on day 35. Despite maximal hemodynamic and metabolic support, the patient remained in a deep coma and ultimately succumbed to irreversible multiorgan failure on day 35 of hospitalization (postoperative day 4).

## Discussion

This case illustrates catastrophic fulminant Cushing’s disease caused by an invasive pituitary macroadenoma. While Cushing’s disease typically presents with insidious, progressive symptoms, a subset of patients present with a rapid and aggressive phenotype [1,2].

Our patient’s presentation, with cortisol levels exceeding 150 µg/dL, ACTH > 200 pg/mL, and life-threatening hypokalemia (1.7 mmol/L), placed her in a high-risk category. The tumor burden was equally critical: an MRI scan revealed an invasive macroadenoma involving the cavernous sinus. Large invasive tumors have historically been associated with lower rates of biochemical remission and higher resistance to medical therapy, setting the stage for the therapeutic challenges encountered [8].

Given the extreme ACTH and cortisol concentrations alongside profound hypokalemia, an ectopic ACTH syndrome was initially considered in the differential diagnosis. A pituitary MRI unequivocally demonstrated an invasive macroadenoma of 28 mm, which pointed to a pituitary origin. In addition, staging thoraco-abdominopelvic CT scans confirmed the absence of neuroendocrine lesions or other sources of ectopic hormone secretion due to occult primary malignancies. The high-dose dexamethasone suppression test showed 58% suppression of serum cortisol. Although this test has limited diagnostic accuracy, particularly in invasive macroadenomas, this result, together with an unequivocal invasive pituitary macroadenoma and negative cross-sectional imaging for an ectopic source, supported the diagnosis of Cushing's disease.

The patient's initial presentation with severe spontaneous mucocutaneous bleeding represents a historically recognized manifestation of severe hypercortisolism. Cortisol excess causes marked thinning of the dermis and subcutaneous connective tissue [9]. The suppression of types I and III collagen production, combined with increased collagen degradation, results in extreme tissue friability and impaired vascular integrity. Additionally, severe chronic hypercortisolemia induces atrophy of perivascular supporting structures, impairs endothelium-mediated vasoreactivity, and dysregulates the nitric oxide/cyclic GMP pathway, culminating in marked microvascular compromise [10,11]. In our patient, this profound microvascular fragility established a prohibitive intraoperative bleeding risk, precluding early transsphenoidal surgical intervention.

Thrombocytopenia is an atypical finding in Cushing's disease, as hypercortisolism typically induces a prothrombotic, hypercoagulable state [3,4]. Isolated reports have described thrombocytopenic purpura in patients with Cushing's syndrome [12] and in the setting of pituitary apoplexy [6], but the mechanisms remain poorly defined. In our patient, platelet counts declined progressively from admission and reached their nadir in the days surrounding the pituitary apoplexy, prompting a systematic diagnostic work-up.

Bone marrow examination showed preserved trilineage hematopoiesis with normal megakaryocytes, excluding a central cause and indicating peripheral destruction. Nutritional deficiency, monoclonal gammopathy, hemophagocytic lymphohistiocytosis, and heparin-induced thrombocytopenia were excluded. The concomitant reticulocytosis, elevated LDH, and undetectable haptoglobin indicated ongoing hemolysis. However, the negative direct antiglobulin test, the absence of schistocytes, and a normal ADAMTS13 activity argued against immune hemolysis, Evans syndrome, and thrombotic thrombocytopenic purpura.

A secondary immune thrombocytopenia was considered, and IVIG was administered. The absence of a platelet response, together with the paradoxical progression of thrombocytopenia, argued against a classic immune mechanism.

Conversely, several findings supported a non-immune consumptive process driven by endothelial activation. Factor VIII (211%), factor V (237%), von Willebrand factor activity (315%), and D-dimer were markedly elevated (3.64 mg/L), reflecting active coagulation and endothelial stress [3,4,13]. There was no evidence of overt disseminated intravascular coagulation: fibrinogen, PT, and aPTT were normal, the ISTH score was 4, and viscoelastic testing was normal. This pattern suggests a platelet-predominant consumption on an activated endothelium, rather than a global consumptive coagulopathy. The mild prolongation of both PFA-100 closure times, obtained at the lower validity limits for platelet count and hematocrit and despite very high von Willebrand factor levels, did not indicate a clinically relevant platelet defect.

Determining causality presents a challenge: did thrombocytopenia precipitate the apoplexy, or did the apoplexy drive the thrombocytopenia? The patient's platelet count at admission was reduced, but the acute, rapidly progressive thrombocytopenia may support the idea that this hemostatic impairment, associated with the fragile tumor vasculature, triggered the intratumoral hemorrhage. However, it is also possible that a subclinical intratumoral infarction or hemorrhage (sentinel apoplexy) initiated a systemic consumption of platelets, creating a vicious cycle that culminated in the acute expansion and cavernous sinus compression [6]. A bidirectional relationship, rather than a single causal direction, therefore appears the most plausible explanation. This "coagulation paradox" serves as a clinical red flag: when a hypercoagulable patient spontaneously develops thrombocytopenia, an acute structural or systemic event, such as tumor necrosis or apoplexy, must be suspected immediately [5].

Another finding was the onset of central DI, requiring desmopressin therapy. In this presentation, DI indicates the interruption of magnocellular neurons in the hypothalamic-neurohypophyseal tract, suggesting severe compression of the pituitary stalk. Furthermore, the manifestation of DI, combined with the subsequent deterioration, strongly raises the suspicion of an early, subclinical "sentinel" apoplexy.

The ACTH peak observed on day 29 (722 pg/mL), coinciding with the apoplectic event, may reflect the release of preformed ACTH from necrotic and hemorrhagic tumor tissue, superimposed on the loss of negative feedback induced by metyrapone. Similar transient hormonal changes may reflect apoplexy of secreting pituitary adenomas, and this finding provides biochemical support for the acute tumor necrosis documented on imaging.

Transsphenoidal surgery remains the gold-standard first-line treatment for Cushing’s disease [7]. However, in this case, immediate neurosurgical intervention was contraindicated due to rapidly progressive thrombocytopenia, metabolic instability, and hemodynamic fragility. Furthermore, the tumor's invasion into the cavernous sinus significantly reduced the probability of a complete surgical cure. Consequently, a decision was made to prioritize medical stabilization to "bridge" the patient to surgery [14]. Unfortunately, rapid neurological deterioration suggested that the tumor’s invasive biology outpaced the window for elective neurosurgical planning.

The medical management followed a logical escalation but faced the limitations of current pharmacotherapy in fulminant cases [14]. Cabergoline is an adjuvant therapy targeting D2 receptors, but it takes time to reach its clinical effect. Mifepristone offers the advantage of rapid peripheral glucocorticoid receptor antagonism without awaiting enzymatic downregulation; however, its use abolished central negative feedback, with a rise in ACTH followed by a marked increase in circulating cortisol. Given the persistent clinical deterioration despite receptor blockade, the therapeutic strategy was shifted to metyrapone, a potent 11-beta-hydroxylase inhibitor. Metyrapone induced a rapid, partial biochemical response, with a fourfold reduction in measured cortisol within four days. As immunoassays cross-react with 11-deoxycortisol during metyrapone therapy, true cortisol levels were probably even lower than those measured. Nevertheless, this biochemical improvement did not translate into clinical control: accumulation of mineralocorticoid precursors (11-deoxycorticosterone) sustained hypokalemia and hypertension [14], and the disease progressed to pituitary apoplexy. This dissociation between biochemical response and clinical failure illustrates the narrow therapeutic window of medical therapy in fulminant corticotroph tumors.

Interpreting circulating cortisol levels during medical escalation poses a major analytical pitfall. Mifepristone competitively blocks glucocorticoid receptors, abolishing central hypothalamic-pituitary-adrenal negative feedback and driving a compensatory rise in ACTH and circulating cortisol. Conversely, metyrapone directly halts steroidogenesis via 11-beta-hydroxylase inhibition, and standard immunoassays may cross-react with the upstream precursor, resulting in overestimation of true active cortisol. For these reasons, treatment response was assessed primarily on clinical and metabolic grounds, worsening vascular fragility, persistent severe hypokalemia, uncontrolled hypertension, and tumoral apoplexy leading to clinical collapse, rather than on measured cortisol alone.

Alternative medical therapies were considered. Mineralocorticoid receptor antagonists (spironolactone or eplerenone) were not initiated because of their delayed antikaliuretic effect relative to the life-threatening hypokalemia. Ketoconazole was avoided because of hepatotoxicity, and mitotane because of delayed onset of action. Osilodrostat and pasireotide were not available in our institutional formulary. Temozolomide was not an option given its myelosuppressive effect in the setting of profound thrombocytopenia, its delayed efficacy, and the absence of histological confirmation. Intravenous etomidate was considered but not used, as the patient was not mechanically ventilated and sedation-related airway compromise was deemed prohibitive given severe bleeding risk. Definitive bilateral adrenalectomy was also prioritized.

With medical therapy failing and the patient deteriorating, bilateral adrenalectomy was performed as a life-saving measure to immediately eliminate the source of cortisol production. This procedure is the definitive treatment for refractory Cushing’s disease but carries significant perioperative mortality [15].

The development of postoperative refractory shock resulted from a complex overlap of mechanisms, including acute withdrawal of endogenous glucocorticoids, surgical stress, and pre-existing critical illness. In fact, the procedure transformed the patient from a state of severe hypercortisolism to absolute adrenal and cortisol insufficiency. Despite stress-dose hydrocortisone, the rapid drop in circulating cortisol could precipitate severe hemodynamic collapse, especially in a body adapted to massive steroid levels.

This case highlights several important clinical lessons. First, severe vascular fragility from hypercortisolism alone can contraindicate surgery, a historically recognized but now rare complication that demands clinical awareness. Second, medical therapy for severe Cushing's disease has significant limitations, particularly in the presence of an aggressive and fulminant presentation. Finally, bilateral adrenalectomy in critically ill patients, while potentially lifesaving, carries an extremely high perioperative mortality rate.

Some limitations inherent to this fulminant presentation must be acknowledged. First, the prohibitive hemorrhagic risk precluded transsphenoidal surgery. Consequently, no pituitary tissue was available for histopathological analysis, proliferation markers (Ki-67, p53), or transcription factor-based classification, and the aggressive nature of the tumor was inferred from its radiological invasiveness and clinical course. The absence of post-mortem examination also precluded histological confirmation of intratumoral necrosis, hemorrhage, and microvascular thrombosis in other organs, which would have supported the proposed consumptive mechanism. Second, as bilateral inferior petrosal sinus sampling was not feasible, the pituitary origin of ACTH secretion was established on the basis of an unequivocal invasive macroadenoma, a suppressive response to high-dose dexamethasone, and the absence of an ectopic source on cross-sectional imaging. Third, the hematological diagnosis remains presumptive: endothelial activation and peripheral platelet consumption were inferred from laboratory markers, without tissue confirmation. Finally, the interpretation of serum cortisol during medical therapy was limited by the compensatory rise in ACTH and cortisol during mifepristone therapy; treatment response was therefore assessed primarily on clinical and metabolic grounds.

## Conclusions

Fulminant Cushing’s disease caused by locally invasive corticotroph macroadenomas represents an exceptional therapeutic challenge, particularly when complicated by atypical and severe hematological derangements. We present a rare case of a pituitary macroadenoma with suspected secondary consumptive thrombocytopenia. This case highlights the necessity of early multidisciplinary risk stratification, vigilant recognition of atypical hematological complications in Cushing's disease, and recognition that multiorgan collapse can occur despite aggressive multidisciplinary interventions.

Further research is needed to understand the mechanisms underlying thrombocytopenia in hypercortisolism and to develop effective strategies for managing such rare but devastating complications. This case contributes to the limited literature on this topic and serves as an instructive example of pituitary adenomas.
